# Quality of Life of asthmatic children and their caregivers

**DOI:** 10.12669/pjms.35.2.686

**Published:** 2019

**Authors:** Nahla Khamis Ibrahim, Maha Alhainiah, Maie Khayat, Orjwan Abulaban, Sarah Almaghrabi, Osama Felmban

**Affiliations:** 1 *Nahla Khamis Ibrahim, Community Medicine Department, King Abdulaziz University, Jeddah, Saudi Arabia. Epidemiology Department, High Institute of Public Health, Alexandria University, Alexandria - Egypt*; 2 *Maha Alhainiah Intern, King Abdulaziz University, Jeddah, Saudi Arabia*; 3 *Maie Khayat Intern, King Abdulaziz University, Jeddah, Saudi Arabia*; 4 *Orjwan Abulaban Intern, King Abdulaziz University, Jeddah, Saudi Arabia*; 5 *Sarah Almaghrabi Intern, King Abdulaziz University, Jeddah, Saudi Arabia*; 6 *Osama Felmban Pediatrics Department, King Abdulaziz University, Jeddah, Saudi Arabia*

**Keywords:** Asthma, Quality of Life, Child, Caregivers, PAQLQ, PACQLQ

## Abstract

**Objectives:**

To assess Quality of Life (QOL), and its associated factors between asthmatic children and their caregivers, and determine the correlation between QOL of patients and caregivers, at King Abdulaziz University Hospital (KAUH), Jeddah.

**Methods:**

A cross-sectional study was conducted among eligible participants who attended Pediatric Pulmonology Outpatient Clinic of KAUH, during 2016/2017. A data collection sheet was used. The standardized Arabic version of Pediatric Asthma Quality of Life Questionnaire (PAQLQ) for children aged 7 - 17 years was completed. The caregiver who accompanied the child fulfilled the Pediatric Asthma Caregiver’s Quality of Life Questionnaire (PACQLQ). Descriptive and inferential analyses were performed.

**Results:**

QOL scores were reduced among asthmatic children who had other type of allergy, or a family history of allergies. Uncontrolled management of asthma presented by frequent waking-up at night, frequent wheezes, visiting Emergency Rooms (ER), or hospital admission was associated with poor QOL of both asthmatic children and their caregivers. There is a positive correlation between child symptoms domain of PAQLQ and emotional domain of PACQLQ of their caregivers.

**Conclusion:**

Uncontrolled asthma was associated with poor QOL of asthmatic child and caregivers. Better management of asthma is recommended to improve their QOL.

## INTRODUCTION

Asthma is a heterogeneous, chronic, complex, inflammatory disease of the respiratory system. It causes recurrent wheezes, chest tightness, dyspnea, and cough. It is the most common chronic disease of childhood and adolescence.^1^ Asthma has big impacts on each of morbidity, mortality and Quality of Life (QOL).^2^-^4^ It was estimated that asthma affected about 7% of adults residing in the USA, and the prevalence is greater between children (about 10%). The Center of Disease Control (CDC) reported that the overall prevalence of lifetime asthma is 10.5%.^5^ As regards Saudi Arabia, a recent study done in Jazan reported that prevalence of asthma among school-children was 10% based on physicians’ diagnosis.^6^ To address the need, manage and control of asthma the Saudi Initiative for Asthma (SINA) has recommended different initiative guidelines for managing the asthma in KSA.^7^

The impact of asthma on the child and their family is far reaching.^8^ Asthma can affect the Quality of Life (QOL) of both children and caregivers.^2^ QOL is usually used in pediatric asthma to determine how asthma affects child’s daily life.^9^ A study from Brazil showed that severity and control of asthma can determine the QOL of asthmatic patients and their families.^4^ However, the factors associated with QoL are not well understood in Jeddah. It is also critical to recognise the relationship between domains of QOL among children and their caregivers, for providing effective management of asthma among children.^10^

The current study was done to assess QOL, and its associated factors between asthmatic children and their caregivers, and to determine the correlation between QOL of asthmatic patients and the caregivers, at KAUH, Jeddah.

## METHODS

A cross-sectional study was conducted over 1-year period (February 2016 - February 2017). Asthmatic children and their caregivers who fulfilled the eligibility criteria were included. The criteria for patient were being an asthmatic child, aged 7-17 years, with or without other atopic conditions (eczema, allergy and rhinitis, conjunctivitis), and attended the Pediatric Pulmonology Outpatient Clinic of KAUH during the study period. The exclusion criteria were presence of other respiratory comorbidity, other chronic medical condition (depression, diabetes mellitus, cancer, etc.), or in case of language barriers.

For each eligible child, one matched main caregiver (father, mother, others) who accompanied the child during the visit to KAUH and accepted to participate in the study was taken. A standardized data collection sheet was used. The face and content validity of the sheet were assessed by two experts. It asked about personal, socioeconomic status, presence of other allergic conditions, and the asthmatic control. The standardized Arabic Version (23 items) of the Pediatrics Asthma Quality of Life Questionnaire (PAQLQ) for children 7-17 years was also used. Furthermore, the Arabic version of Pediatric Asthma Caregiver’s Quality of Life Questionnaire (PACQLQ) was used. The standardized Arabic versions of both questionnaires have good validity and reliability.^11^ Validity and reliability of the adapted Arabic translation of PAQLQ-A was assessed among Egyptians.^12^

Data analysis was conducted using the SPSS version 21. All calculations of PAQLQ and PACQLQ were done. Descriptive and inferential statistics were calculated. Student’s *t*–test, and Pearson’s correlation were performed. All *P-value*s ≤ 0.05 were considered statistically significant.

The study followed the ethical standards of “Declaration of Helsinki”. The study was approval by the Research Ethics Committee (REC) of KAUH, with a Reference Number of 403-15. Approval for using the standardized Arabic version of the PAQLQ and the PACQLQ was taken from the author.

## RESULTS

A total of 61 children (7-17 years old), and *61* caregivers fulfilled the eligibility criteria, and completed the study. The mean age (SD) of asthmatic children was 9.48 (2.73) years, with a male to female ratio of 1:1.18.

About one-half (49.2%) of the asthmatic patients had family history of allergies, and 67.2% of them complained from other allergic disorder (beside asthma). Rhinorrhoea, allergic conjunctivitis and sinusitis were reported by 39.3%, 16.4% and 8.2% of asthmatic children, respectively.

At least one trigger of asthma was identified among the majority (95.1%) of asthmatic children. Table-I. The most frequently reported triggers were exposure to fumes (80.3%), weather changes (78.7%), and perfumes (45.9%).

**Table-I:** Triggers of asthma exacerbation among asthmatic children attended Paediatric Pulmonology Clinic at in King Abdulaziz University Hospital.

Triggers	No.	%
Identified asthma triggers (≥ 1)	58	95.1
Fumes	49	80.3
Weather changes	48	78.7
Perfumes	28	45.9
Dusts	24	39.3
Pesticides	23	37.7
Exercise	16	26.2
Cats	16	26.2
Anxiety or nervousness	13	21.3
Humidity	12	19.7
Detergents	11	18.0
Cosmetics	10	16.4
Grass	3	4.9
Dogs	1	1.6
Fungi	1	1.6
Leaves	1	1.6

Every trigger was separately asked about.

Gender didn’t significantly affect QOL of the asthmatic children. Table-II. The total QOL score was lower among asthmatic children who had family history of allergy compared to others (Student’s t= 2.46, *P* < 0.05). Children who complained from other allergic disorder(s) had significantly (*P*< 0.01) lower mean scores of all QOL domains compared to the counterpart children without additional allergies.

**Table-II:** Factors affected quality of life of asthmatic children who attended Pulmonology Clinic at King Abdulaziz University Hospital.

	Quality of life domains							

Variables	Physical activity		Symptoms		Emotional		Total score	

	Mean	(SD)	Mean	(SD)	Mean	(SD)	Mean	(SD)
Gender:								
Male (n=33)	26.45 (7.57)		52.96 (14.45)		46.21 (12.17)		125 (26.28)	
Female (n=28)	27.78 (6.29)		57.46 (11.45)		48.64 (9.58)		133 (32.33)	
Student’s t test, p	0.0738	.463	1.32	.189	0.855	.396	1.081	.284
Family history of allerg y:								
Yes (30)	25.53 (7.42)		50.17 (14.85)		44.57 (12.90)		120.27 (33.62)	
No (31)	28.54 (6.32)		59.74 (9.56)		50.0 (8.23)		138.29 (22.65)	
Student’s t test, p	-1.71	.09	-3.00	.004	-1.97	.05	-2.46	.01
Presence of other allergies:								
Yes	20.22 (8.45)		41.88 (17.88)		38.00 (16.20)		38.00 (16.20)	
No	28.25 (6.05)		57.30 (10.98)		48.94 (9.17)		48.94 (9.17)	
Student’s t test, p	-3.45	.001	-3.51	.006	-2.91	.001	-3.51	.001
Wheezes (6 months):								
< 3 times	28.41 (5.96)		57.94 (10.51)		49.72 (8.44)		136.07 (23.07)	
≥ 3 times	16.71 (5.79)		32.57 (10.73)		28.85 (11.79)		78.14 (26.21)	
Student’s t test, p	4.89	.000	5.99	.000	5.87	.000	6.16	.000
Wake up at night (symptoms)								
< 3 times	28.16 (6.38)		57.14 (11.63)		49.16 (8.90)		134.5 (25.18)	
≥ 3 times	22.09 (7.76)		45.45 (16.34		39.0 (15.77		106.5 (38.77)	
Student’s t test, p	2.74	0.008	2.79	0.002	2.93	0.005	2.99	0.004
Inhaled corticosteroid								
Yes	23.21 (7.73)		48.63 (15.32)		40.58 (14.07)		112.42 (36.11)	
No	28.81 (5.93)		57.92 (11.24)		50.38 (7.79)		137.12 (22.99)	
Student’s t test, p	-3.09	.003	-2.66	.01	-3.50	.001	-3.23	.002
O_2_ nebulizer (6 months):								
Yes	24.57 (8.19)		52.39 (15.05)		43.26 (13.34)		120.2 (35.39)	
No	28.58 (5.75)		56.63 (11.96)		49.78 (8.67)		135.0 (24.62)	
Student’s t test, p	-2.06	.02	-1.22	.29	-2.32	.02	-1.92	.05
Hospital admission (6 months):								
Zero -2 times	25.55 (7.42)		50.16 (14.85)		44.56 (12.89)		33.62 (6.13)	
≥ 3 times	28.54 (6.31)		59.74 (9.56)		50.00 (8.23)		22.65 (4.06	
Student’s t test, p	-1.71	0.092	-3.00	.004	-1.69	.054	-2.46	.017
ER visits (last 6 month s):								
Never	30.84 (4.19)		61.47 (6.81)		54.11 (2.55)		146.42 (12.29)	
≥ 1 times	25.36 (7.36		52.11 (14.45)		44.26 (12.02)		121.74 (32.17)	
Student’s t test, p	3.02	.004	2.69	0.009	3.52	.001	3.27	.002
Skipping school:								
Yes	26.85 (7.35)		54.48 (13.68)		46.68 (11.43)		128.0 (31.01)	
No	28.71 (2.69)		59.28 (8.78)		52.28 (5.61)		140.0 (14.34)	
Student’s t test, p	-0.660	.512	-0.901	.371	-1.270	.209	-1.027	.309

All QOL domains (physical, symptoms, emotional) were significantly better between asthmatic children who didn’t visit emergency room (ER) during the six months preceded the study (*P* < 0.01). Asthmatic children who suffered from ≥ 3 attacks of wheezy chest (during the six months preceded the survey) had significantly much worse values of the three QOL domains compared to others (*P* < 0.001). Children’s QOL scores were significantly lower also among those who reported frequent waking-up at night (due to symptoms as cough) compared to others. Children received inhaled corticosteroids or oxygen nebulizer obtained also much lower QOL scores than others.

Regarding QOL of caregivers of asthmatic children, Table-III illustrates that there is no statistical significant association between PACQLQ domains and gender of caregiver, or age of the asthmatic child. PACQLQ domains were lower among caregivers who had more than one asthmatic children, but without statistical significance (*P* > 0.05). The scores of emotional function and physical activity domains were better among caregivers who reported that their children wake-up were less frequently during night due to cough. A similar decrease in QOL was observed among caregivers whose children received inhaled corticosteroids compared to others (P > 0.05). Furthermore, the score of total QOL domain was lower among caregivers whose children reported more frequent visits of the ER unit than others. The mean score of the emotional function domain of PACQLQ was significantly higher among the caregivers whose children reported ≥ 3 times of hospital admission during the 6 months preceded the study compared to others (Student’s t= 2.33, *P* < 0.05). Similar finding were also observed regarding the total PACQLQ domain and the number of hospital admission.

**Table-III:** Factors affected quality of life of caregivers who accompanied asthmatic children at King Abdulaziz University Hospital.

Quality of life domain Variable	Emotional function		Physical activity		Total score	

	Mean	SD	Mean	SD	Mean	SD
Gender of caregiver						
Female	31.86 (12.27)		10.93 (6.39)		42.79 (17.74)	
Male	31.69 (8.04)		12.36 (5.30)		44.06 (12.46)	
Student’s t test, p	0.061	0.95	-0.959	.34	-0.328	0.74
Age of child						
≤ 9	33.61 (8.69)		11.67 (5.52)		45.28 (13.20)	
>9	29.12 (11.53)		11.66 (6.33)		40.88 (17.21)	
Student’s t test, p	1.734	.08	-.060	.95	1.12	0.26
Having other children:						
Yes	31.60 (9.09)		11.55 (7.05)		43.33 (15.68)	
No	31.79 (10.27)		13.4 (.7.05)		45.00 (.15.68)	
Student’s t test, p	0.04	0.96	0.67	0.50	0.24	0.81
Number of asthmatic children						
1 child	33.27 (9.39)		12.16 (5.54)		45.43 (13.67)	
2 or more	29.45 (10.93		11.00 (6.28)		40.46 (16.69)	
Student’s t test, p	1.452	0.15	0.759	0.45	1.272	0.20
Child wake up at night (symptoms)						
< 3 times	32.78 (9.76)		12.26 (5.76)		45.04 (14.42)	
≥ 3 times	27.18 (10.87)		9.18 (5.65)		36.36 (16.18)	
Student’s t test, p	1.68	0.09	1.61	0.11	1.77	0.08
corticosteroidReceived inhaled						
Yes	30.59 (9.69)		10.78 (5.28)		41.37 (13.99)	
no	33.06 (10.57)		12.72 (6.29)		45.79 (15.95)	
Student’s t test, p	-0.95	0.34	-1.13	0.19	-1.15	0.25
Received oxygen nebulizer						
Yes	34.91 (12.61)		12.43 (7.24)		47.34 (19.06)	
no	29.64 (7.86)		11.26 (4.80)		41.13 (11.55)	
Student’s t test, p	1.93	0.06	0.76	0.45	1.59	0.11
ER visit (6 months)						
< 3 times	32.76 (10.34)		12.46 (5.67)		45.23 (15.14)	
≥ 3 times	28.43 (8.82)		9.14 (5.74)		37.57 (13.32)	
Student’s t test, p	1.42	0.16	1.91	0.06	1.71	0.09
No. of admission (6 months)						
< 3 times	32.61 (9.78)		12.18 (5.76)		44.80 (14.54)	
≥ 3 times	24.0 (10.58)		7.33 (4.76)		31.33 (14.67)	
Student’s t test, p	2.33	0.04	1.98	0.05	2.15	0.03

Presence of significant positive correlations between domains of PAQLQ with each other’s was seen. Table-IV. For example, PAQLQ activity limitation domain had strong positive correlation with the patient’s symptoms and patient’s emotion (*p* < 0.001). Similar findings were observed regarding PACQLQ. Emotional domain of PACQLQ had positive correlation with the domain of patients’ symptoms of the PAQLQ.

**Table-IV:** Correlations between quality of life domains of asthmatic children and their caregivers at Pulmonology Clinic, King Abdulaziz University Hospital.

Domain	Emotional Domain of caregiver	Activity domain of caregiver	Child activity	Child symptoms	Child emotions
Emotional domain of caregiver	1	0.754^**^	0.226	0.256^*^	0.203
Activity of caregiver		1	0.165	0.187	0.133
Child activity			1	.827^**^	.812^**^
Child symptoms				1	.891^**^
Child emotions					1

** *P* < 0.001,

* *P* < 0.05

## DISCUSSION

In the current study, asthmatic children who complained from other allergic disorder(s) had significantly lower scores of all PAQOL domains compared to others. Similarly, a study from Scotland reported that presence of rhinitis beside asthma affected QOL of the asthmatic children.^13^

At least one asthma trigger was identified among the majority of asthmatic children, which agrees with Cabana, et al.^14^ from the USA. The most frequently reported triggers were the exposure to fumes, weather changes and perfumes. On the other hand, Cabana’s study showed the majority of triggers were related to plants, animals, dust, weather and smoke. These differences could be related to time of conduction of both study.^14^

In the current study, asthmatic patients who reported more frequent attacks of wheezy chest, admissions to hospital, visiting ER, using of oxygen nebulizers, or inhaled corticosteroids obtained lower QOL scores than others. Such findings suggested that QOL is related to the level of the asthma control, which coincides with another study concluded that severity of asthma was linked to child QOL.^9^ Similarly, a recent Indian study, 2018, revealed better presence of QOL between children with controlled asthma.^15^ Results from a systematic review concluded that children whose asthma symptoms are not well-controlled were more likely to have impairment QOL.^16^ The study of Scotland also showed that severity of asthma was one of the predictors of child QOL.^13^ Similarly, Al-Gewely, et al.^17^ evaluated QOL of Egyptian asthmatics using PAQLQ and found that those who obtained higher scores had better asthma control.

Understanding factors affecting QOL of the caregivers is important in managing asthma. This will be helpful to provide better condition to control asthma.^18^ In the present study, caregivers of children with controlled asthma had better PACQLQ scores compared to others, which agrees with the recent Indian study.^15^

Children’s symptoms of PAQLQ was significantly associated with emotional PACQLQ domain of their caregivers. This indicates that quality of control asthmatic patients significantly impacts the emotions of their caregivers. Similar result was reported from Atlanta Empowerment Zone.^18^ These findings are also in line with results of two studies from Poland.^19^-^20^

## CONCLUSIONS

About one-half of the asthmatic patients in the current study had family history of allergic diseases, and about one fifth of them had other allergic condition(s) beside asthma. The most frequent reported asthma triggers were exposure to fumes, weather changes and perfumes. Asthma affects QOL of the asthmatic children and their caregivers. Children whose asthma symptoms are not well-controlled were more likely to have impairment of their QOL. This uncontrolled management of asthma manifested by frequent waking up at night, wheezing, and frequents visits to ER were associated with poor QOL of both asthmatic child and caregivers. The emotional domain of the caregivers was statistically associated with the patients’ symptoms. Proper patients’ and parents’ education about asthma, and good control would provide better QOL for both groups. Further studies regarding guidelines & asthma control are recommended to improve the QOL of asthmatic children and their caregivers.
